# Pregnancy in Patients With Renal Transplant

**DOI:** 10.1097/og9.0000000000000169

**Published:** 2026-05-14

**Authors:** Ibrahim Elali, Corrine K. Nelson, Linda M. Szymanski, Adley I. Lemke, Samy M. Riad, Randall J. Hinojosa, Andrea G. Kattah

**Affiliations:** Division of Nephrology, Hypertension, and Transplantation, Department of Medicine, UCLA David Geffen School of Medicine, Harbor-UCLA Medical Center, and the Lundquist Institute, Torrance, California; and the Division of Maternal-Fetal Medicine, Department of Obstetrics and Gynecology, the Division of Nephrology and Hypertension, Department of Medicine, and the Department of Pharmacy, Mayo Clinic, Rochester, Minnesota.

## Abstract

Pregnancy after kidney transplantation is increasingly common. Maternal and fetal outcomes are generally favorable with stable allograft function and individualized care. Current guidelines recommend waiting at least 1 year posttransplant before attempting pregnancy to ensure graft stability and reduce the risk of complications. Prepregnancy counseling is essential and should include information about transitioning to a compatible immunosuppressive regimen to minimize teratogenic risks. Pregnancy management in transplant recipients requires a multidisciplinary approach involving the transplant clinician, transplant pharmacist, and maternal–fetal medicine specialist. This review highlights key considerations and clinical recommendations across each phase of pregnancy care: prepregnancy planning, pregnancy management, and postnatal follow-up, emphasizing the importance of collaborative, patient-centered strategies for reproductive health in kidney transplant recipients.

## Abstract 2

Risks to mother and fetus can be minimized in patients with kidney transplant when the pregnancy is planned and monitored through a multidisciplinary approach.

Reproductive-age patients with advanced chronic kidney disease (CKD), including those on dialysis, may regain hormonal and ovulatory function after kidney transplantation.^1^ By the time women reach CKD stage 5 or begin dialysis, more than 90% experience irregular or absent menstrual cycles, which is often attributed to disruption of the hypothalamic-pituitary-gonadal axis by uremic toxins and hyperprolactinemia.^2^ Return of ovulation can occur as soon as 3 weeks after transplantation, although more typically within 6–12 months posttransplant.^1^

The first reported successful pregnancy after kidney transplant was in a 23-year-old woman in March 1958, who had received a kidney from her identical twin 2 years prior.^3^ She delivered two full-term neonates by cesarean . Since then, more than 14,000 pregnancies have been documented in renal allograft recipients worldwide.^4^

In general, planned posttransplant pregnancy does not appear to increase the rate of graft loss or allograft rejection. However, it is associated with increased maternal and fetal morbidity,^5^ albeit outcomes in kidney transplant recipients are comparable with those in patients with CKD with similar degrees of kidney function who have not received kidney transplant.^6^ Although previous data suggested that serum creatinine level of greater than 1.4 mg/dL is associated with an increase in adverse pregnancy outcomes, several factors, including the etiology of kidney disease, presence of hypertension, and proteinuria, may further mediate risk.^6–8^ Pregnancy timing should be individualized for each patient, with factors including graft function stability and maternal age taken into account.^8^ Generally, pregnancy should be deferred for at least 1 year after transplantation^9^ and up to 2 years when feasible.^10^

In this review, we divide pregnancy care into three sections: prepregnancy, during pregnancy, and postpregnancy. Generally, pregnancy after kidney transplantation is associated with better maternal and fetal outcomes compared with pregnancy in patients with advanced CKD or end-stage renal disease (ESRD).^11^

## PREPREGNANCY

### Family Planning

Unintended pregnancy after kidney transplantation can pose risks to the fetus and mother, as well as the survival of the allograft^12^ (Appendix 1, available online at http://links.lww.com/AOG/E649). Previously, the rate of posttransplant planned pregnancies in the United States, as reported in the University of Nebraska and the Mayo Clinic cohorts, ranged from 71% to 87% respectively.^13,14^ Contraception counseling is recommended, and we generally suggest long-acting reversible contraception options, such as intrauterine devices (IUD) and subdermal implants (etonogestrel implants). Historically, there have been concerns regarding the use of IUDs in kidney transplant recipients due to the risk of contraceptive failure and intra-abdominal infections, but subsequent studies in other immunocompromised population showed IUD use to be safe (Appendix 2, available online at http://links.lww.com/AOG/E649).^15^ In patients with solid organ transplants without graft failure, there are no restrictions on either hormonal or nonhormonal IUDs. In patients with graft failure, the advantages of IUD placement generally outweigh the potential risks.^16^ Other options for contraception include injective contraceptives such as depo-medroxyprogesterone acetate, oral contraceptive pills, and barrier methods. The latter are considered the least effective for contraceptive purposes and require the concurrent use of two methods per the Risk Evaluation and Mitigation Strategy, a mandatory, clinician-assisted drug safety monitoring program managed by the U.S. Food and Drug Administration (FDA).

The effects of medications on male fertility, sexual function, and sperm parameters (count, motility, and morphology) should be considered during family planning after transplantation. We recommend transitioning male transplant recipients off mammalian target of rapamycin (mTOR) inhibitors at least 3 months before the time of desired pregnancy as a means of reversing the significant decrease in spermatogenesis caused by these agents. No change is recommended for calcineurin inhibitors, antimetabolites, or corticosteroid immunosuppression^17–24^ (Appendix 3, available online at http://links.lww.com/AOG/E649).

### Prepregnancy Counseling on Kidney and Pregnancy Outcomes

Numerous studies have documented favorable kidney allograft outcomes after pregnancy.^5,25,26^ However, these outcomes are predominantly reported in patients with preserved graft function, typically with prepregnancy mean serum creatinine levels ranging from 1.2 to 1.4 mg/dL. As such, caution should be exercised when extrapolating these results to patients with lower estimated glomerular filtration rate (eGFR)^5,25,26^ (Appendix 4, available online at http://links.lww.com/AOG/E649). A recent Dutch cohort study evaluating 288 pregnancies in 192 women after kidney transplantation found that *adverse pregnancy outcomes*—defined as low birth weight (LBW), preterm delivery, severe hypertension, or a 15% or greater rise in serum creatinine—were predictive of subsequent graft loss (hazard ratio 2.55; 95% CI, 1.09–5.96). However, this association lost statistical significance after adjusting for prepregnancy eGFR, underscoring the pivotal role of baseline renal function in predicting pregnancy-related graft outcomes. Notably, only 10 patients in this study had an eGFR below 30 mL/min/1.73 m^2^, highlighting the need for further research in this high-risk, understudied population.^8^

The live-birth rate in pregnancies among kidney transplant recipients is 70–75%, higher than that in the general population. This is partially attributed to better pregnancy monitoring and planning, yet rates of fetal comorbidities such as LBW and preterm birth (before 37 weeks of gestation) are significantly higher among transplant recipients compared with the general population (Table 1).^5,26^ With regard to maternal health, the risks of cesarean delivery and preeclampsia are significantly higher in transplant recipients compared with the general population, with preeclampsia risk ranging from 20–30% compared with 2–5% in the general population.^26^ The long-term effects of preeclampsia on renal allograft function remain unclear (Appendix 5, available online at http://links.lww.com/AOG/E649).^27^

**Table 1.** Comparison of Pregnancy Outcomes in Kidney Transplant Recipients and the U.S. General Population*

Outcome	U.S. General Population^85,86^	Kidney Transplant Recipients^5,8^
Live-birth rate (%)	62	72.9–93.0
Miscarriage (%)	10–20	14.0–15.4
Stillbirth (%)	0.5	0.9–5.1
Preterm delivery (%)	10.4	43.1–59.5
Gestational age at delivery (wk)	39	34.9–35.6
Mean birth weight (g)	3,332	2,383–2,470
Preeclampsia (%)	4.6	21.5–48.6

* U.S. data are from the National Vital Statistics System, Natality and Fetal Death public-use files, National Center for Health Statistics, Centers for Disease Control and Prevention. CIs provided when available.

### Immunosuppression Considerations in Pregnancy Planning

Before pregnancy, teratogenic medications must be replaced with safe alternative agents; we recommend that all medications must achieve therapeutic stability with stable allograft function (Table 2), for at least 3 months. We also recommend starting intake of prenatal multivitamins containing at least 400 micrograms of folic acid.^28^

**Table 2.** Summary of Immunosuppressive Medications, Pregnancy Considerations, and Monitoring Recommendations

Medication	Pregnancy Considerations	Monitoring
Tacrolimus (calcineurin inhibitor)	• Benefits generally outweigh risks • Dose often increased 20–100% due to enhanced metabolism during pregnancy • Risk of elevated unbound levels in anemia or hypoalbuminemia	• Trough levels: monthly (1st and 2nd trimester), every 2 wk in 3rd trimester • Lower target acceptable if albumin <3 g/dL or RBC count <3.5 million/microliter
Cyclosporine (CNI)	• Benefits generally outweigh risks • May require 20–25% dose increase	• Monthly trough levels throughout pregnancy • Adjust dose to maintain prepregnancy target
Azathioprine (antimetabolite)	• Safe alternative to mycophenolate mofetil; minimal transfer to fetus • Check thiopurine methyltransferase transferase before starting Transition at least 6 wk before pregnancy, ideally 3 months	• Routine renal and liver laboratory tests • Watch for cytopenias
Mycophenolate mofetil (antimetabolite)	• Requires REMS program • Contraindicated in pregnancy due to risk of miscarriage and birth defects • Transition to azathioprine at least 6 wk prepregnancy	• Monitor graft function after switch to alternative agents
Prednisone (corticosteroid)	• Benefits may outweigh risks • Slight risk of orofacial clefts; inconsistent data • Stress-dose steroids recommended during delivery if on chronic use	• Continue maintenance dosing • Monitor for gestational diabetes and hypertension
mTOR inhibitors (sirolimus, everolimus)	• Limited data; concerns about fetal toxicity and impaired wound healing • Recommend stopping at least 3 mo prepregnancy	• Monitor graft function after switch to alternative agents
Belatacept	• Limited data; may be continued in select cases • Consider transition to CNI-based regimen prepregnancy • Increased volume of distribution may require dose adjustment	• Monitor closely if continued; not first-line • Monitor graft function after switch to alternative agents

RBC, red blood cell; CNI, calcineurin inhibitor; REMS, Risk Evaluation and Mitigation Strategy; mTOR, mammalian target of rapamycin.

#### Tacrolimus

Increased metabolic enzyme expression, increased blood volume, and decreased drug binding protein expression change tacrolimus pharmacokinetics, with peak effect occurring at around 26–28 weeks of pregnancy.^29^ The result is an increased unbound fraction of tacrolimus and an increased whole-blood oral clearance. Decreases in albumin, alpha-1 acid glycoprotein, and red blood cells lower whole-blood tacrolimus levels but do not affect unbound tacrolimus concentrations.

If no other factors alter unbound tacrolimus concentrations, the unbound fraction of tacrolimus remains consistent during pregnancy despite decreases in whole-blood tacrolimus levels.^29^ Based on available data, a 20–100% increase in calcineurin inhibitor dose is required during pregnancy to maintain prepregnancy and postpartum whole-blood levels, but achieving these levels in patients with hypoalbuminemia (albumin less than 3 g/dL) and anemia (red blood cell count less than 3.5 million/microliter) significantly increases the unbound fraction of tacrolimus.^29^ The unbound fraction of tacrolimus is not measurable in clinical practice, and clinical outcomes associated with higher unbound concentrations are unavailable.^30^ Given this complexity of tacrolimus kinetics and monitoring, we recommend allowing tacrolimus levels to be permissively lower in pregnant patients with hypoalbuminemia and anemia to minimize tacrolimus-related adverse effects from elevated exposure.

#### Cyclosporine

Cyclosporine primarily binds to lipoproteins. Pregnancy-related changes in lipoproteins are mainly unknown, making it difficult to predict changes in unbound cyclosporin concentration during pregnancy.^30^ We recommend continuing cyclosporine therapy to achieve prepregnancy trough goals while monitoring and managing medication-related adverse effects^31^ (Appendix 6, available online at http://links.lww.com/AOG/E649).

#### Mycophenolate and Azathioprine

Postmarketing reports of increased structural malformations in children of transplant recipients maintained on mycophenolate mofetil led the FDA to establish the Risk Evaluation and Mitigation Strategy program to prevent pregnancies in patients taking mycophenolate.^32,33^ Subsequent studies have confirmed the risk of malformation and have added a higher rate of miscarriage, preterm births, and LBW with exposure to mycophenolate in pregnancy.^32,34,35^

Azathioprine is generally considered safe for transplant recipients during pregnancy,^36,37^ and at lower doses very little azathioprine or 6-mercaptopurine is transferred across the placenta and into fetal circulation.^37^ Additionally, the fetus lacks the enzymes responsible for converting azathioprine to its active metabolites.^38^ Package inserts and the Risk Evaluation and Mitigation Strategy program both recommend transition from mycophenolate to azathioprine at least 6 weeks before pregnancy.^33,34^ However, we recommend an earlier transition to provide at least 3 months of monitoring for allograft stability before pregnancy; we also consider adding prednisone maintenance therapy for patients previously on a corticosteroid-free maintenance immunosuppression regimen if they are deemed a higher risk of rejection. Thiopurine methyltransferase enzyme activity testing is recommended before pregnancy to guide azathioprine dosing. In male users, a theoretical teratogenic effects from mycophenolate use exists and is reflected in recommendations by Semet et al^21^. However, available evidence suggests that pregnancy outcomes involving fathers who are transplant recipients on mycophenolate are similar to those of the general population.^20,21,39,40^

#### Prednisone

Prednisone has been included in transplant recipient immunosuppression regimens, since the first reports of kidney transplant.^41–43^ Maternal corticosteroid use during early pregnancy has been inconsistently associated with orofacial clefts in offspring.^44–46^ Steroid use may negligibly increase the risk of cleft lip or cleft palate or both from a baseline risk of 1.0–2.0 per 1,000 to 1.2–3.4 per 1,000. Maintaining immunosuppressive doses of prednisone does not add significant risks, and we recommend continuing with prepregnancy dosing (Appendix 7, available online at http://links.lww.com/AOG/E649).

#### Mammalian Target of Rapamycin Inhibitors

Limited data are available on mTOR inhibitors, sirolimus, and everolimus, as they are less frequently used for immunosuppression maintenance. There are reports of sirolimus and everolimus use throughout pregnancy; however, there are concerns regarding fetal toxicity (mortality and delay in ossification).^47–50^ There are generally concerns regarding toxicity with these drugs in pregnancy, as opposed to teratogenicity.^51,52^ Due to concerns for fetal toxicity, higher rates of cesarean delivery in transplant recipients, and additional risks of delayed wound healing, we recommend transitioning off mTOR inhibitors at least 3 months prepregnancy to monitor graft function stability and regimen tolerability.

#### Belatacept

Little information is available concerning the use of belatacept during pregnancy.^53,54^ The biggest case series to date, by Coscia et al,^55^ showed favorable fetal outcome and no birth effect (Appendix 8, available online at http://links.lww.com/AOG/E649). If belatacept is continued during pregnancy, it is anticipated that dose increases will be required due to increases in blood volume and, thus, volume of distribution.^56^ We have recommended continuing belatacept in selected pregnancy cases despite the limited data, because pregnancy outcomes with belatacept appear to be comparable with those with calcineurin inhibitors, such as in women with a history of thrombotic microangiopathy attributed to calcineurin inhibitors.

## DURING PREGNANCY

In pregnancy, a multidisciplinary approach is highly recommended for kidney transplant recipients. The multidisciplinary team includes maternal–fetal medicine specialists, the transplant team, pharmacists, and dieticians.

We recommend ultrasonography around 6–8 weeks of gestation to confirm pregnancy viability and estimated delivery date. Because beta-HCG is partially cleared by the kidneys (20–30%),^57^ serum false positives can be high. After confirmation of viability and gestational age, a discussion with the patient regarding detailed first-trimester ultrasonography and genetic screening should be undertaken. A detailed first-trimester ultrasonogram, completed between 11 0/7 and 13 6/7 weeks of gestation, can be offered to evaluate for abnormalities (increased nuchal translucency, significant fetal anomalies). Furthermore, the American College of Obstetricians & Gynecologists recommends that every pregnant person be offered genetic screening, diagnostic testing, or both if applicable during pregnancy.^58^ Noninvasive prenatal testing (NIPT) is a prenatal aneuploidy screening test (typically screening for chromosomes 13, 18, and 21) that detects fetal cell-free DNA in maternal blood samples. It is considered the most sensitive and specific screening test for common fetal aneuploidies and can be performed as early as 10 weeks of gestation and until delivery. Data on NIPT in the transplant population are limited; however, the accuracy of NIPT for the common trisomies appears to be high.^59,60^ Importantly, a current recommendation is to not evaluate sex chromosomes in this population because the results will be affected by the sex of the organ donor.^59,60^ Additionally, if any genetic screening returns as “high risk,” patients should be referred for further discussion of diagnostic testing via chorionic villus sampling or amniocentesis, depending on gestational age.

An advanced-level fetal anatomy survey is recommended between 18 and 20 weeks of gestation, in addition to serial fetal growth ultrasonograms every 3–4 weeks starting around 24 weeks. In certain situations, fetal echocardiography may be indicated between 22 and 24 weeks of gestation.

Screening for gestational diabetes, a condition more common in kidney transplant recipients, is typically completed between 24 and 28 weeks of gestation.^61^ In addition to routine prenatal care, fetal growth assessments and antenatal fetal surveillance should be individualized. We recommend beginning serial ultrasonograms for fetal growth in the third trimester and initiating weekly antenatal surveillance via nonstress test or a biophysical profile at 32 weeks of gestation. If there are any additional complications during pregnancy (ie, fetal growth restriction or maternal hypertension), earlier and more frequent testing may be indicated.^62^

Delivery timing is dependent on multiple factors, including the presence of hypertension, fetal growth restriction, obstetric history, and maternal status throughout pregnancy. If there are no complications, our general practice is to recommend delivery at between 37 and 39 weeks of gestation, favoring the later end of this range.^4^

Evidence on the optimal mode of delivery in renal transplant recipients is limited. Expert consensus recommends that cesarean delivery be reserved for standard obstetric indications, although existing studies demonstrate higher cesarean delivery rates in kidney transplant recipients.^63^ A 2019 meta-analysis by Shah et al^5^ reported a cesarean delivery rate of approximately 62% among kidney transplant recipients. Importantly, a large cohort study by Yin et al, comparing trial of labor with scheduled cesarean delivery found that in kidney transplant recipients, attempting labor if appropriate was associated with improved neonatal outcomes without an increase in severe maternal morbidity.^64,65^

There are no formal guidelines for cesarean delivery technique in renal transplant recipients. The transplanted kidney is typically located in the iliac fossa, low and anterior in the pelvis, with the ureter implanted into the bladder. Skin incision may need to be modified (eg, midline vertical rather than Pfannenstiel), and creation of a bladder flap is generally avoided to protect the ureter. Surgeons should also use caution with retractor placement and limit uterine exteriorization to minimize potential graft trauma.^66^

It is common for kidney transplant patients to require steroids during pregnancy. If patients are taking prednisone (or its equivalent) dosing of 10 mg/day or more for 3 or more weeks, stress dose steroids should be considered during delivery (Appendix 9, available online at http://links.lww.com/AOG/E649).

### Hypertension Management

The threshold for diagnosis of hypertension in pregnancy is blood pressure higher than 140/90 mm Hg.^67^ The 2021 Kidney Disease Improving Global Outcomes (KDIGO) clinical practice guidelines recommend a BP control goal of lower than 130/80 mm Hg in patients with CKD.^68^ The Society for Maternal-Fetal Medicine recommends a blood pressure goal of 130/80 or lower in renal transplant recipients with chronic hypertension. Our general recommendation is to have a target BP of 130–140/80–90 mm Hg during pregnancy in kidney transplant recipients.^67,69,70^ We also recommend home blood pressure monitoring.

Antihypertensive therapy during pregnancy in patients with kidney transplant is similar to that in the general pregnant population, in which labetalol and nifedipine extended release are considered first-line medications. Methyldopa can be safely used during pregnancy; however, it appears to be less effective and is poorly tolerated.^71^ Angiotensin-converting enzyme inhibitors and angiotensin receptor blockers, if used for renoprotection, should be discontinued at the time of pregnancy due to the risk of fetotoxicity, specifically renal toxicity, and the risk of stillbirth.^68^

Patients with a history of chronic hypertension have an increased risk of developing preeclampsia. Guidelines recommend that women at high risk of preeclampsia take low-dose aspirin between week 12–16 to reduce their risk, and to continue through delivery.^72,73^ In renal transplant recipients, diagnosis can be challenging^71,74^ (Appendix 10, available online at http://links.lww.com/AOG/E649). Hyperuricemia is not a helpful marker because calcineurin inhibitors can increase blood uric acid levels via decreased urate clearance, although the overall incidence is higher in patients treated with cyclosporine.^75,76^

The FDA has approved the use of angiogenic markers, specifically the ratio of serum soluble fms-like tyrosine kinase 1 to placental growth factor (sFlt-1:PlGF), to help diagnose preeclampsia in patients between 23 and 35 weeks of gestation with hypertensive disorders. When used with other laboratory tests and clinical assessments, the sFlt-1/PlGF ratio can aid in evaluating the risk of developing preeclampsia with severe features within 2 weeks. In patients in this population with no history of CKD a value less than 40 has a high negative predictive value.^77^ This ratio and placental growth factor alone have been used for ruling out preeclampsia in patients with kidney transplant,^78^ with promising results, but this method is not yet the standard of care.^79^

### Immunosuppression Management

We typically measure tacrolimus trough monthly in the first and second trimesters, then every 2 weeks starting at 28 weeks of gestation unless more frequently needed. Table 3 summarizes our recommended blood tests schedule and calcineurin inhibitor monitoring during pregnancy.

**Table 3.** Suggested Laboratory and Clinical Visit Schedule

Laboratory Test	1st Trimester (wk 0–13)	2nd Trimester (wk 14–26)	3rd Trimester (wk 27–40)
Renal function panel (fasting)	Monthly	Monthly	Every 2 wk
CBC with differential	Monthly	Monthly	Every 2 wk
Tacrolimus	Monthly levels* Trough goal^†^	Monthly levels* Trough goal^†^	Every 2 wk levels* Trough goal^†^
Cyclosporine	Monthly levels* Trough goal^†^	Monthly levels* Trough goal^†^	Monthly levels* Trough goal^†^
UA, Ucx, UPCR/UACR^‡^	Monthly	Monthly	Every 2 wk
BK/CMV/Toxo PCR	If clinically indicated	If clinically indicated	If clinically indicated
LFTs	Once	Monthly	Every 2 wk
Nephrology visit	Monthly	Monthly	Monthly

CBC, complete blood count; UA, urine albumin; Ucx, urine culture; UPCR, urine protein to creatinine ratio; UACR, urine albumin to creatinine ratio; BK, BK virus; CMV, cytomegalovirus; Toxo, *Toxoplasma gondii*; PCR, polymerase chain reaction; LFTs, liver function tests.

* More frequently for cause or monitoring dose adjustments.

† Maintain the prepregnancy tacrolimus/cyclosporine goal with the same albumin and RBC levels. Lower tacrolimus trough levels are acceptable when serum albumin is less than 3 g/dL or RBC count is less than 3.5 (10^12^/L) or both. If the tacrolimus trough level is less than 4.5, increase tacrolimus dose by 25% and recheck level in 1 week.

‡ Consider weekly home urine dipstick testing starting in the second trimester if there is no preexisting albuminuria. If indicated, 24 urine samples (protein, albumin, sodium, and creatinine clearance) are considered at the end of the first trimester and every trimester thereafter.

### Transplant Surveillance

Donor-derived cell-free DNA testing for rejection surveillance during pregnancy is contraindicated according to the manufacturer's instructions and has not yet been validated for use in kidney transplant recipients.^79^ Because there is not a noninvasive test for rejection available for pregnant patients, even if preeclampsia can be ruled out with angiogenic markers, a biopsy may still be needed in select cases.

Should it be clinically indicated, a biopsy is generally considered safe based on anecdotal reports and is preferably performed before 26–28 weeks of gestation. Some studies have reported a high risk of bleeding around 23–26 weeks of gestation in native kidney biopsy during pregnancy.^80,81^ However, several recent case series have highlighted the safety and therapeutic importance of kidney biopsy in pregnancy.^82,83^ As always, risks and benefits need to be weighed carefully for kidney biopsy.

## AFTER DELIVERY

### Immunosuppression Management

Tacrolimus or cyclosporine trough levels are checked at 2 weeks and 4 weeks after delivery, monthly for 3 months, and then resumed testing per the center protocol. Pregnancy hormone–related induction of drug-metabolizing enzymes may take up to 3 or 4 months to stabilize, and close monitoring of tacrolimus during that time is recommended as the anticipated dose decreases.^84^ We continue tacrolimus, azathioprine, and prednisone 5 mg daily (if used) throughout the breastfeeding period. If no further pregnancies are planned and appropriate contraception is initiated, we transition patients to mycophenolate mofetil, with no required overlap period. We also consider checking anti human leukocyte antigen antibodies 1–3 months after delivery.

## CONCLUSION

**Fig. 1.**
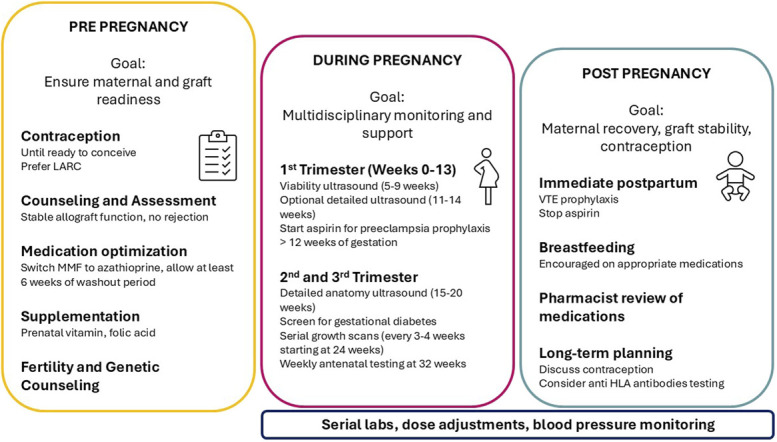
Summary of pregnancy planning recommendations in the kidney transplant recipient.

Pregnancy in kidney transplant recipients can be successful with careful interdisciplinary planning and follow-up. Several studies have shown favorable allograft outcomes after pregnancy, though most patients included in those series have had preserved eGFR, which appears to be one of the most important factors in adverse pregnancy outcomes and graft failure. Establishing a protocol of a minimum frequency of laboratory testing and clinic visits can help both patients and clinicians navigate pregnancy and the postpartum period successfully (Fig. 1, Box 1). Data on kidney transplantation in pregnancy are limited; many recommendations for the pregnant kidney transplant recipient are extrapolated from the non–kidney transplant pregnant patient or based on expert opinion to guide therapy in this special patient population.

*Elali. Pregnancy in Patients With Renal Transplant. O&G Open 2026*.

Box 1. **Clinical Pearls and Suggestions: Pregnancy Decision Should be Individualized**

- Stable graft function, minimum 1 y, 2 y is optimal
- Serum creatinine <1.4 mg/dL preferred
- Proteinuria <500–1,000 mg/d preferred
- Age consideration for the mother
- No recent rejection or infection for at least 1 y before pregnancy
- Controlled medical conditions, ie, hypertension, diabetes mellitus, risk of underlying disease recurrence
- Access to a multidisciplinary care team, including the transplant team, maternal–fetal medicine specialists, pharmacists, and dieticians
